# Supplementary material for: Examining recent trends in the racial disparity gap in tap water consumption: NHANES 2011–2018
Source: Public Health Nutr. 2021 Jun 11;25(2):207–13. doi: 10.1017/S1368980021002603 (PMC8664888; doi:10.1017/S1368980021002603)
Supplement: Supplementary file 1 [file S1368980021002603sup001.docx]

**Supplemental Materials for**

**Title: Examining Recent Trends in the Racial Disparity Gap in Tap Water Consumption: NHANES 2011–2018**

**Supplemental Table 1:** Log-binomial regression models of not drinking tap water by survey cycle, race/ethnicity, and socio-demographics, NHANES 2011-2018 among children/adolescents and adults.

|  | Adjusted Prevalence ratio of not drinking tap water | Adjusted Prevalence ratio of not drinking tap water |
| --- | --- | --- |
|  | (95% CI) | (95% CI) |
| Independent variables | Children/adolescents | Adults |
|  | Model 1 | Model 2 |
| Survey cycle 2011-12 | 1.00 | 1.07 |
|  | (0.76 - 1.32) | (0.84 - 1.37) |
| 2013-2014 | 1 | 1 |
| 2015-16 | 1.28* | 1.17 |
|  | (0.96 - 1.70) | (0.96 - 1.43) |
| 2017-18 | 1.63*** | 1.40*** |
|  | (1.25 - 2.12) | (1.16 - 1.69) |
| Race/ethnicity: NH white | 1 | 1 |
| NH Black | 2.46*** | 2.28*** |
|  | (1.79 - 3.38) | (1.96 - 2.65) |
| NH Asian | 1.84*** | 1.49*** |
|  | (1.35 - 2.52) | (1.22 - 1.80) |
| Hispanic | 3.12*** | 1.89*** |
|  | (2.41 - 4.03) | (1.56 - 2.29) |
| Other/Mixed | 1.52** | 1.52*** |
|  | (1.04 - 2.20) | (1.26 - 1.84) |
| Age: ^a^2-5 years / ^b^60+ | 1 | 1 |
| 6-11 years / 20-39 | 0.88** | 1.01 |
|  | (0.78 - 1.00) | (0.90 - 1.14) |
| 12-19 years / 40-59 | 0.50*** | 1.10* |
|  | (0.44 - 0.58) | (0.99 - 1.22) |
| Nativity status: Born in US | 1 | 1 |
| Born outside US | 1.49*** | 1.53*** |
|  | (1.23 - 1.80) | (1.31 - 1.80) |
| Male | 1 | 1 |
| Female | 1.10* | 1.22*** |
|  | (0.99 - 1.22) | (1.13 - 1.32) |
| ^c^FIPR >350% | 1 | 1 |
| ≤130% | 1.42** | 1.23** |
|  | (1.06 - 1.90) | (1.05 - 1.45) |
| 131-350% | 1.33* | 1.15* |
|  | (0.98 - 1.81) | (1.00 - 1.34) |
| Education^b^: College + | -- | 1 |
| Less than high school | -- | 2.12*** |
|  |  | (1.77 - 2.53) |
| High school grad | -- | 1.93*** |
|  |  | (1.60 - 2.33) |
| Some college | -- | 1.61*** |
|  |  | (1.37 - 1.89) |
| HH reference education^a^: College + | 1 | -- |
| Less than high school | 1.55*** | -- |
|  | (1.19 - 2.00) |  |
| High school grad/some college | 1.42*** | -- |
|  | (1.10 - 1.84) |  |
|  |  |  |
| Observations | 9,439 | 17,268 |

95% CI in parentheses; *** p<0.01, ** p<0.05, * p<0.1;

^a^for children; ^b^for adults;

^c^FIPR: federal income to poverty ratio; household income for children;

Note: Data using question inquiring about normal drinking water source in which persons indicated if they did not consume their tap water.

**Supplemental Table 2:** Estimated amount of people in the US not drinking tap water and change between 2013-2014 and 2017-2018.

|  | 2013-2014 | 2017-2018 | Total change |
| --- | --- | --- | --- |
| Children/adolescents (2-19 year olds) | 9,167,905 | 14,822,954 | 5,655,049 |
| Adults (20+ years) | 33,184,518 | 46,553,820 | 13,369,302 |
| Total | 42,352,423 | 61,376,774 | 19,024,351 |

Note: Data calculated from adjusted prevalence estimates in this paper multiplied by the NCHS census population totals (https://wwwn.cdc.gov/nchs/nhanes/responserates.aspx#population-totals) in 2013-14 and 2017-18.
